# Human intrahepatic ILC2 are IL-13^positive ^amphiregulin^positive^ and their frequency correlates with model of end stage liver disease score

**DOI:** 10.1371/journal.pone.0188649

**Published:** 2017-12-19

**Authors:** Hannah C. Jeffery, Patrick McDowell, Philipp Lutz, Rebecca E. Wawman, Sheree Roberts, Chris Bagnall, Jane Birtwistle, David H. Adams, Ye Htun Oo

**Affiliations:** 1 Centre for Liver Research and National Institute for Health Research Birmingham Biomedical Research Centre, Institute of Immunology and Immunotherapy, University of Birmingham, Birmingham, United Kingdom; 2 Department of Internal Medicine I, University of Bonn, Bonn, Germany; 3 School of Life Sciences, Faculty of Health and Life Sciences, Coventry University, Coventry, United Kingdom; 4 Human Biomaterial Resource Centre, University of Birmingham, Birmingham, United Kingdom; 5 Clinical Immunology Department, University of Birmingham NHS Foundation Trust, Birmingham, United Kingdom; 6 University Hospital of Birmingham NHS Foundation Trust, Birmingham, United Kingdom; McGill University, CANADA

## Abstract

**Introduction:**

Innate lymphoid cells (ILC) have been implicated in the initiation of inflammation and fibrosis in mice. However, ILC have not been characterized in inflamed human liver tissue.

**Methods:**

Human intrahepatic lymphocytes were isolated by mechanical digestion and phenotyped by flow cytometry. Conditioned medium from cultures of primary human biliary epithelial cells, stellate cells, fibroblasts and inflamed human liver tissue was used to model the effects of the inflammatory liver environment of ILC phenotype and function.

**Results:**

All three ILC subsets were present in the human liver, with the ILC1 (CRTH2^neg^CD117^neg^) subset constituting around 70% of intrahepatic ILCs. Both NCR^pos^ (NKp44^+^) and NCR^neg^ ILC3 (CRTH2^neg^CD117^pos^) subsets were also detected. ILC2 (CRTH2^pos^) frequency correlated with disease severity measured by model of end stage liver disease (MELD) scoring leading us to study this subset in more detail. ILC2 displayed a tissue resident CD69^+^ CD161^++^ phenotype and expressed chemokine receptor CCR6 allowing them to respond to CCL20 secreted by cholangiocytes and stellate cells. ILC2 expressed integrins VLA-5 and VLA-6 and the IL-2 and IL-7 cytokine receptors CD25 and CD127 although IL-2 and IL-7 were barely detectable in inflamed liver tissue. Although biliary epithelial cells secrete IL-33, intrahepatic ILC2 had low expression of the ST2 receptor. Intrahepatic ILC2 secreted the immunoregulatory and repair cytokines IL-13 and amphiregulin.

**Conclusions:**

Intrahepatic ILC2 express receptors allowing them to be recruited to bile ducts in inflamed portal tracts. Their frequencies increased with worsening liver function. Their secretion of IL-13 and amphiregulin suggests they may be recruited to promote resolution and repair and thereby they may contribute to ongoing fibrogenesis in liver disease.

## Introduction

Innate lymphoid cells (ILCs) have recently been identified in both humans and mice. ILCs differentiate from the common lymphoid progenitor (CLP), lack antigen-specific receptors and depend on IL-2Rγc signaling. Three groups of ILCs (ILC1, ILC2, ILC3) have been described that share some biological activities with T helper (Th1) (ILC1), Th2 (ILC2) and Th17/22 (ILC3) subsets. ILCs are identified by lack of known lineage markers associated with T cells, B cells, myeloid cells, or granulocytes, but share expression of the common γ chain, IL-7Rα (CD127) and IL-2Rα (CD25)[1]. When activated ILCs secrete cytokines and thereby contribute to the immediate, first-line immune reaction against infection and tissue damage.

ILCs are enriched at barrier surfaces such as the skin, lung and intestine where they function as first responders to environmental antigens by rapidly secreting pro-inflammatory and immune-regulatory cytokines [2–5]. Human ILC1 have been described at inflamed mucosal surfaces in the gut, lung and skin [6]. Type 2 ILC (ILC2) are characterized by expression of CRTH2 and CD161 [7] and respond to PGD_2_ [8] and alarmins including IL-25 and IL-33 by releasing IL-4, IL-5, IL-13 [9, 10]. In the intestine, ILC2 regulate the influx of tissue-resident eosinophils through IL-13 and IL-5 [11]. Amphiregulin, a member of the epidermal growth factor family, which mediates tissue repair, has been reported on murine respiratory and human dermal ILC2 [12, 13], [14]. ILC2 have been implicated in murine liver fibrosis [15, 16] and biliary repair [17].

The human liver receives 75% of its blood from the gut via the portal vein. The terminal branches of the portal vein end close to bile ducts in the portal tract, which can also contain viable bacteria if the intestinal barrier is breached [18, 19]. The biliary epithelial lining of the intrahepatic bile ducts provides defense against gut-derived bacteria entering the liver via bile [20]. Thus the portal tracts include important mucosal surfaces that provide both a physical barrier and a prompt immune response to protect the systemic circulation from gut-derived infection. In this report, we describe the ILC subsets in human liver and suggest that ILC2 in particular play an important role in the local immune response at the level of the intrahepatic bile ducts.

## Materials and methods

### Liver specimens

Explanted diseased liver was obtained from patients undergoing liver transplantation for inflammatory liver diseases including primary biliary cirrhosis (PBC), primary sclerosing cholangitis (PSC), alcoholic liver disease (ALD), non-alcoholic steatohepatitis (NASH) and autoimmune hepatitis (AIH). Normal liver (NL) was obtained from donor liver tissue surplus to clinical requirements or from uninvolved liver tissue removed at the time of resection for colorectal hepatic metastases. All samples were collected with appropriate patient consent and Black Country research ethics committee approval (REC ref. CA/5192; Q2708). None of the transplant donors were from a vulnerable population and all donors or next of kin provided written informed consent that was freely given.

### Blood specimens

Peripheral blood samples were collected in EDTA from individuals with haemochromatosis (taken as control) and the autoimmune liver disease (AILD) autoimmune hepatitis (AIH). All samples were collected with appropriate patient consent and Black Country research ethics committee approval (REC ref. CA/5192; Q2708 or REC 13–150).

### Isolation of human intrahepatic lymphocytes

Intrahepatic lymphocytes were prepared and isolated from fresh liver tissue as previously described [21].

### Isolation of peripheral blood mononuclear cells (PBMC)

Peripheral blood mononuclear cells were isolated from the whole blood by density gradient separation using Lympholyte (VH Bio Ltd) at 800 x *g* for 20 minutes. The mononuclear layer was collected and washed twice with Phosphate Buffered Saline.

### Phenotypic analysis of human intrahepatic and peripheral blood ILC subsets by multicolour flow cytometry

Freshly isolated lymphocytes were stained on ice with antibodies against functional surface markers of interest together with antibodies to identify ILC subsets including: CD3 (UCHT1, BioLegend), CD45 (5B1, Miltenyi Biotec), CD127 (eBioRDR5, eBiosciences) and lineage markers (CD1a (HI149), CD11c (MJ4-27G12), CD14 (TUK4), CD16 (VEP13), CD19 (LT19), CD34 (AC136), CD94 (REA113), CD123 (AC145), BDCA2 (AC144), FceR1 (CRA1) (all from Miltenyi Biotec)) to identify total ILC, CRTH2 (BM16) and CD117 (104D2) (both from BioLegend) to distinguish ILC1 (CRTH2-CD117-), ILC2 (CRTH2+CD117+/-) and ILC3 (CRTH2-CD117+). Antibodies to functional surface markers included: NKp44 (P44-8) and CXCR3 (GO25H7) (both from BioLegend), ST2 (FAB5231P, R and D Systems), CCR6 (11A9) and CD25 (MA-251) (both from BD Biosciences), CD69 (FN50), VLA5/CD49s (NKI-SAM1), VLA6/CD49f (GoH3) and CD161 (191B8) (all from Miltenyi Biotec). For analysis of Amphiregulin, cells surface stained for ILC markers were fixed, permeabilised and stained with anti-Amphiregulin (G-4, Santa Crutz) using the FOXP3/Transcription Factor Staining Kit by eBiosciences according to manufacturers instructions. For intracellular cytokine analysis, cells were cultured at 2.5x10^6^cells/ml in RPMI medium supplemented with Penicilin (100 IU/ml), Streptomycin (100 IU/ml), Glutamine (2 mM) (all from Gibco) and 10% heat-inactivated Foetal Bovine Serum (FBS) (Sigma-Aldrich) and activated with Phorbal Myristate Acetate (PMA) (2.5ng/ml, Sigma-Aldrich) and ionomycin (0.5μM, Sigma-Aldrich) for 4.5 hours. Brefeldin A (5μg/ml, Sigma-Aldrich) was added to the cultures for the final 4 hours to block cytokine secretion. Cells were stained on ice for surface markers, then fixed at room temperature in 3% formaldehyde solution for 20minutes. After washing twice with PBS, cells were permeabilised on ice in prechilled permeabilisation buffer (0.5% Triton X-100, 5% FBS) for 30minutes before adding antibodies against intracellular cytokines including IFN-γ (4S.B3, eBioscience), IL-4 (8D4-8, BD Bioscience), IL-5 (JES1-39D10) and IL-13 (JES10-5A2) (both from BioLegend) at 4°C for 30minutes. In all analyses, dead cells were excluded by staining with the Zombie NIR^TM^ fixable viability dye (BioLegend,) prior to surface staining. Data were acquired using a CyAn flow cytometer and analysed using FlowJo software version 10 (Tree Star Inc).

### Isolation and culture of primary human biliary epithelial cells

Primary human biliary epithelial cells (BEC) were prepared from fresh liver tissue as previously described [21], [22, 23]. BEC were stimulated with IFN-γ (10ng/ml, Peprotech) + TNF-α (10ng/ml, Peprotech) or lipopolysaccharide (LPS) (1μg/ml, Sigma-Aldrich) for 24 hours.

### Isolation of primary human stellate cells and fibroblasts

Primary human stellate cells and fibroblasts were prepared from fresh liver tissue as previously described [24].

### Generation of inflamed human liver supernatant

Liver supernatants were prepared by culturing liver tissue in RPMI medium supplemented with Penicilin (100 IU/ml), Streptomycin (100 IU/ml), Glutamine (2 mM) (all from Gibco) at 1g tissue/1ml RPMI overnight. Liver supernatants were filtered and remaining debris removed by centrifugation before freezing at -20°C or below.

### Luminex analysis of human inflamed liver supernatant and primary cell culture supernatants

IL-2, IL-7, IFN-γ, IL-9, IP-10, IL-25 and IL-33 were analysed by Luminex assays (Bio-Rad). PGD2 level was measured by ELISA (Cambridge Biosciences).

### Detection of amphiregulin expression with immunohistochemistry

Amphiregulin (G-4, Santa Cruz) and CD3 (Abcam) immunostaining in human liver was conducted on paraffin embedded tissue and imaged with a VECTRA slide scanner.

### Statistical analysis

Differences between two disease categories (normal liver vs. diseased liver) were evaluated by Mann-Whitney test and between multiple disease categories (including: normal, Autoimmune Immune Liver Disease (PSC, PBC and AIH) and ALD/NASH by Kruskal Wallis test followed by Dunn’s multiple comparison tests. Data are presented as median ± interquartile range. The effect of a treatment on a certain cell type was tested by Paired *t*-test following confirmation of normality by Kolmogorov-Smirnov test. Data are presented as mean ± standard error of the mean (SEM). Correlations between subset frequencies and clinical parameters were tested by Spearman’s rank correlation test. All analyses were performed using GraphPad Prism 5.0 software (GraphPad software, San Diego, CA, USA). *P* < 0.05 was considered as statistically significant.

## Results

### ILC subsets are present in both normal and inflamed human liver

**Fig 1:**
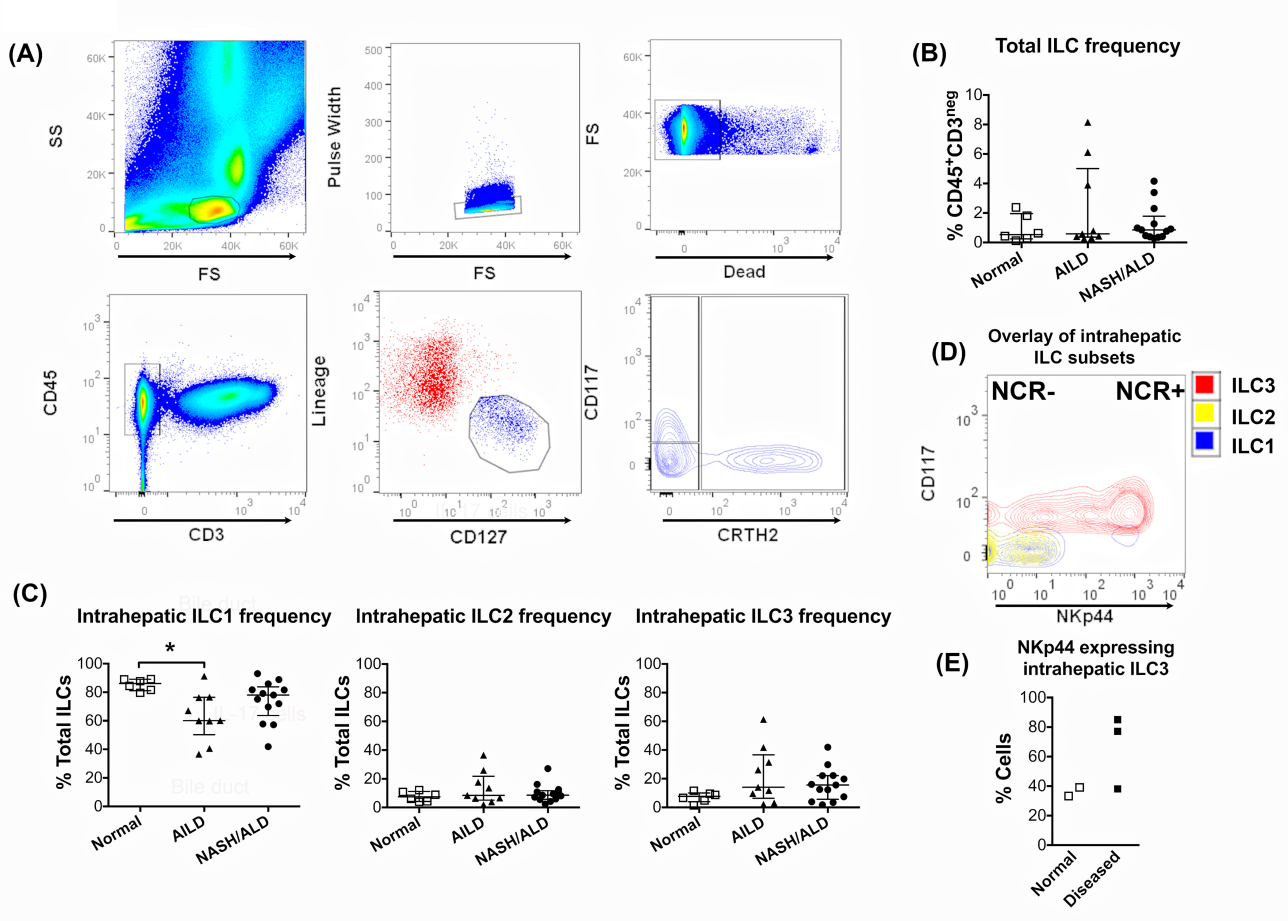
All three ILC subsets are present in inflamed human livers. (A) ILC gating strategy. Intrahepatic lymphocytes were freshly isolated and gated on the CD45^pos^ CD3^neg^ population. The total ILC population was defined as lineage^neg^ and CD127^pos^. Of these, ILC1 were defined as CD117^neg^CRTH2^neg^, ILC2 as CD117^pos/neg^CRTH2^pos^ and ILC3 as CD117^pos^ CRTH2^neg.^ (B) Frequency of the total ILC population among the CD3^neg^ CD45^pos^ population. (C) Frequencies of the three major ILC subsets in the total intrahepatic ILC in different inflammatory liver diseases. AILD: Autoimmune Liver Diseases (PSC, PBC, AIH); NASH: Nonalcoholic Steatohepatitis; ALD: Alcoholic Liver Disease. (* = *p*<0.05 by Kruskal Wallis test). (D) Distribution of NKp44 expression among intrahepatic ILC. ILC3 were NKp44^pos^ (NCR^pos^) or NKp44^neg^ (NCR^neg^). (E) Frequencies of NKp44 expression by intrahepatic ILC3 in normal and diseased livers. Summary data are median ± Interquartile range.

We investigated the frequency of human intrahepatic ILCs in both diseased and normal human liver (Fig 1). ILC were gated as shown in Fig 1A. All three subsets ILC1, ILC2 and ILC3 were detected in human liver tissue at a frequency of between 0.2 and 8% of CD45^+^CD3^neg^ intrahepatic lymphocytes (Fig 1B). Type 1 innate lymphoid cells (ILC1) comprised the majority of intrahepatic ILCs (Fig 1C). ILC1 frequencies were significantly reduced in autoimmune liver disease compared with normal liver tissue and also showed tendency for reduction in the non-autoimmune liver diseases. Trends toward increased frequencies of ILC2 and ILC3 cells were also seen with disease. Two distinct subsets of ILC3 were detected based on NKp44 expression (NKp44^pos^ (NCR^pos^) and NKp44^neg^ (NCR^neg^)) (Fig 1D). Comparison of NKp44 expression by ILC3 from diseased and normal livers suggested that there is induction of NKp44 in the inflamed, fibrotic diseased liver (Fig 1E).

### Intrahepatic ILC2 frequencies correlate with MELD score

**Fig 2:**
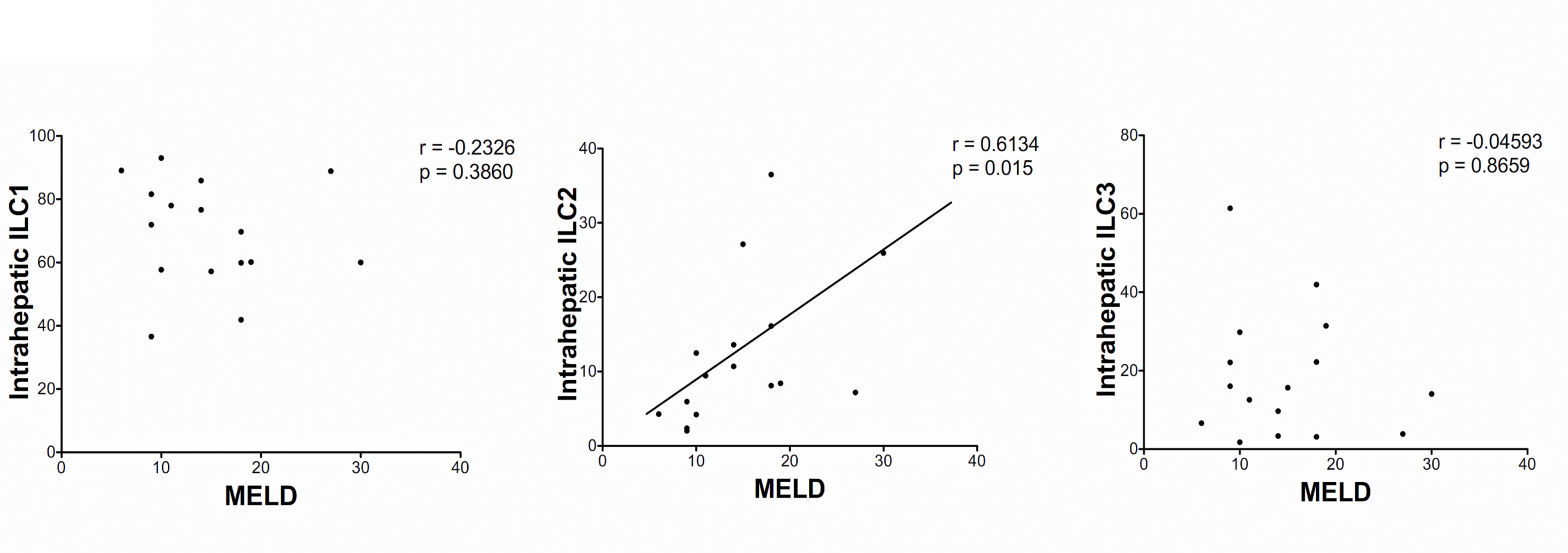
ILC2 subset correlates with MELD score. Correlation between MELD score and frequency of intrahepatic ILC subsets. Correlation of ILC subset frequency with MELD score was assessed by Spearman’s rank correlation test. End-stage livers of all types of inflammatory liver disease (autoimmune, and non-autoimmune) were included in this analysis. Model of End-stage Liver Disease (MELD).

In contrast to ILC1 or ILC3, the frequency of human intrahepatic ILC2 cells correlated with severity of liver disease (Fig 2). In view of this correlation we chose to focus our subsequent investigations on the ILC2 subset.

### Intrahepatic CRTH2^pos^ ILC2 show a tissue resident phenotype and express CD161

**Fig 3:**
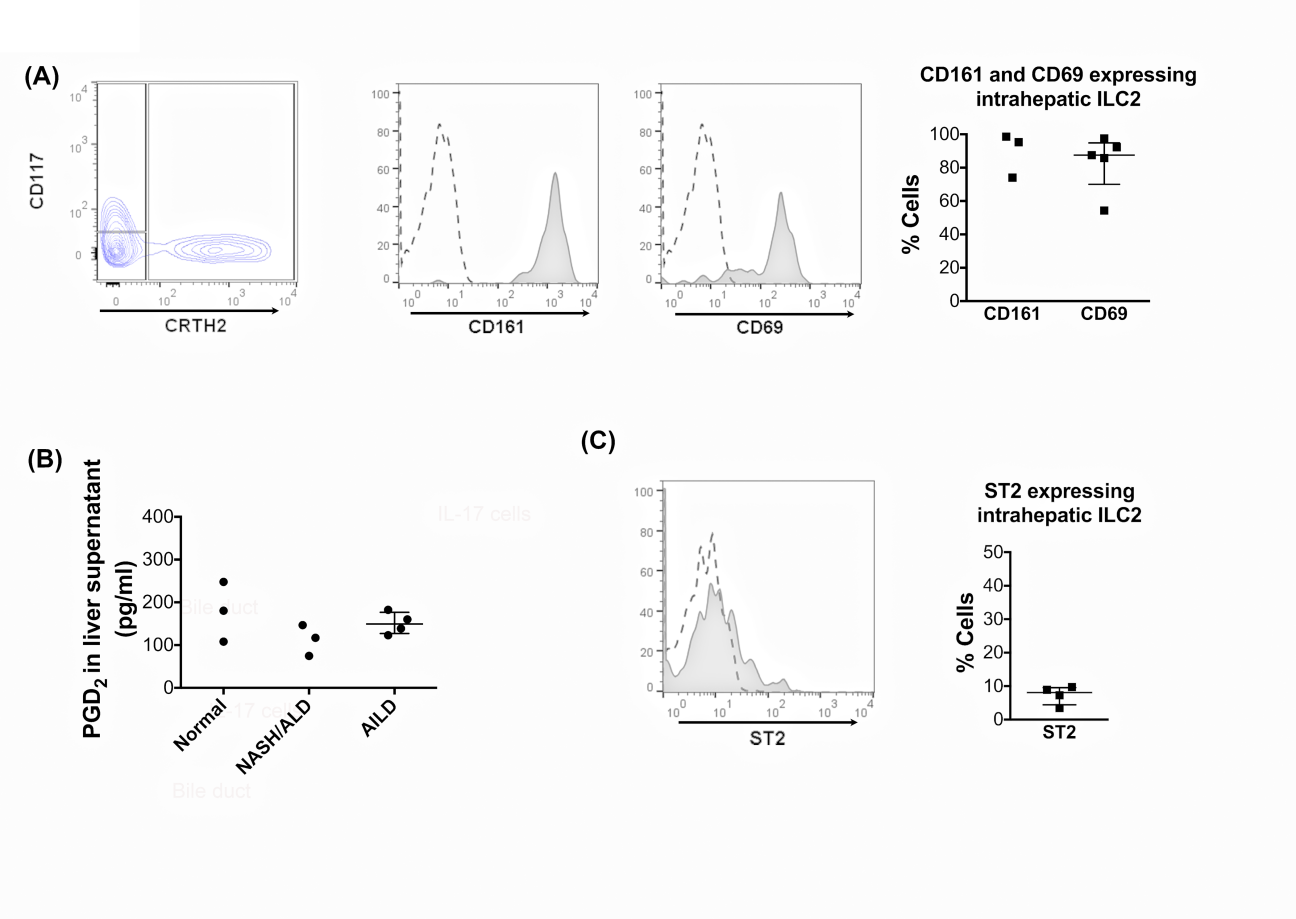
Intrahepatic human ILC2 display a tissue resident phenotype, express CD161 and PGD2 is present in human inflamed livers. (A) The CD45^pos^ CD3^neg^ lineage^neg^ CD127^pos^ CRTH2^pos^ ILC2 subset was gated and CD161 and CD69 expressions were analysed. CD161 and CD69 representative overlays and summary data are shown. (B) PGD2 production by human liver. The secretion of PGD2 by normal and inflamed human liver tissue was analysed by ELISA on 24-hour liver tissue supernatants prepared for 1g-tissue/1ml culture medium. Summary data are median ± Interquartile range. (C) Expression of the IL-33 receptor, ST2 was analysed on ILC2. Representative overlay and summary data are shown. In histogram overlays, dotted lines are isotype staining and shaded histograms marker expression.

ILC2 constitutively expressed CD161 and the tissue residence marker, CD69 (Fig 3A). The ILC2-defining receptor, CRTH2 is the receptor for PGD_2_ and we confirmed the presence of PGD_2_ in liver tissue by measuring levels in the supernatants from cultured liver tissues. PGD_2_ was secreted by both diseased and non-diseased liver tissue (Fig 3B). We detected the IL-33 receptor, ST2, on less than 10% of intrahepatic ILC2 (Fig 3C).

### CD127^pos^ ILC2 express high levels of CD25 although the liver contains little of their cognate cytokines IL-7 and IL-2

**Fig 4:**
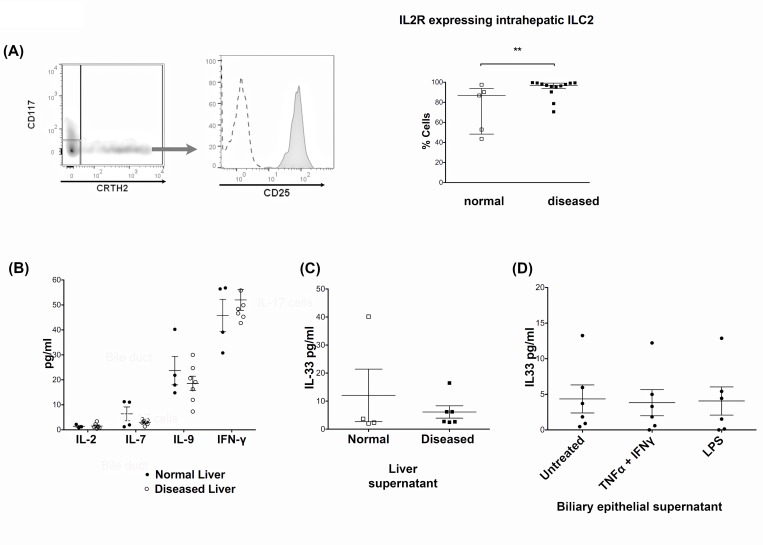
CD127^pos^ ILC2 express a high level of CD25, but inflamed human liver supernatant contains minimal IL-2 and IL-7. (A) The CD45^pos^ CD3^neg^ lineage^neg^ CD127^pos^ CRTH2^pos^ ILC2 subset was gated and expression of the IL-2 receptor α-chain, CD25, was analysed. CD25 representative overlay and summary data in normal and diseased livers is shown. (** = *p*<0.01 by Mann Whitney test). In the histogram overlay the dotted line represents isotype staining and the shaded histogram reports CD25 staining. (B) Human inflamed liver supernatant was analysed for IL-2, IL-7, IL-9 and IFN-γ. The secretion of cytokines by normal and inflamed human liver tissue was analysed by luminex of 24-hour liver tissue supernatants prepared for 1g of tissue/1ml culture medium. (C) IL-33 production by human liver tissue (Normal and diseased) and (D) IL-33 production by Primary human biliary epithelial (BEC). IL-33 in 24-hour supernatants generated by 1g liver tissue/1ml medium or BEC cells unstimulated, stimulated with IFN-γ and TNF-α or with lipopolysaccharide (LPS) was analysed by ELISA. Summary data are median ± Interquartile range.

All ILC subsets are characterised by expression of the IL-7 receptor, CD127. ILC2 also highly expressed the IL-2α receptor, CD25 (Fig 4A). IL-9 has been reported to be a survival factor for ILC2 while IFN-γ inhibits ILC2 function [25, 26]. These observations lead us to measure these cytokines in conditioned supernatants from human liver tissue cultures. We detected only minimal levels of IL-2 and IL-7, moderate levels of IL-9 but high levels of IFN-γ (Fig 4B), suggesting that diseased liver tissue constitutes an unfavourable environment for ILC2. ILC have been reported to respond to IL-33 [9, 10], an alarmin cytokine secreted by epithelial cells. Thus we analysed IL-33, but detected low levels in liver tissue supernatants (Fig 4C) and both un-stimulated and stimulated BEC supernatants (Fig 4D). This corresponded with the low frequency of IL-33 receptor expressing intrahepatic ILC2 that we observed (Fig 3C).

#### Expression profiles of integrins and chemokine receptors on ILC2

**Fig 5:**
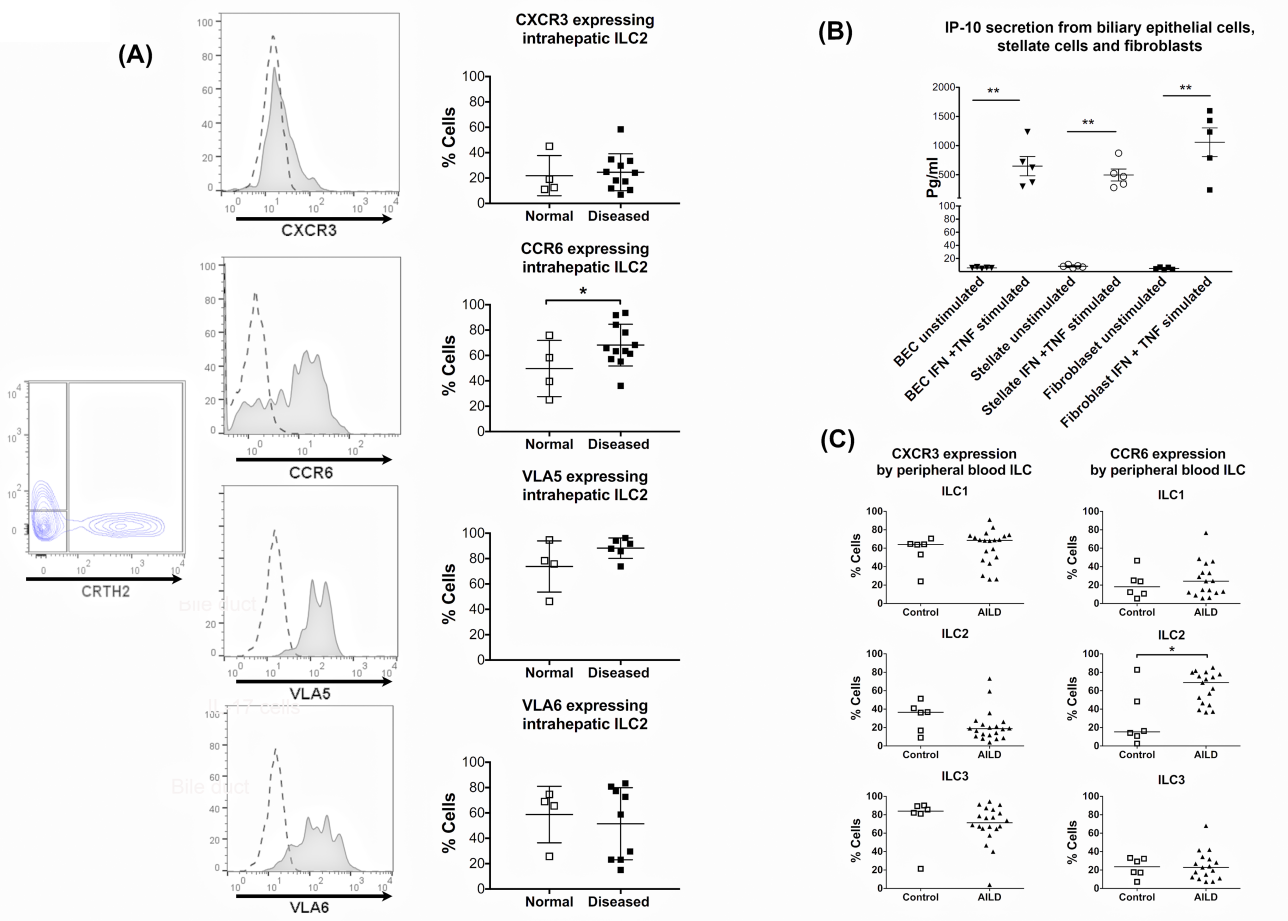
Intrahepatic ILC2 highly express biliary tropic chemokine receptor CCR6 and integrins for fibronectin and laminin. (A) The CD45^pos^ CD3^neg^ lineage^neg^ CD127^posi^ CRTH2^pos^ ILC2 subset was gated and expressions of CXCR3, CCR6, Very Late Antigen-5 (VLA-5) and Very Late Antigen-6 (VLA-6) were analysed. Representative overlays and summary data in normal and diseased livers are shown (normal livers = open squares; diseased livers = filled squares). Summary data are median ± Interquartile range. In histogram overlays, dotted lines represent isotype staining and shaded histograms show the marker expression. (B) Primary human biliary epithelial cells (BEC), stellate cells and fibroblasts were isolated and stimulated with IFN-γ and TNF-α. Interferon gamma-induced Protein-10 (IP-10) secretion was analysed by ELISA. (* = p<0.05, ** = <0.01 by Paired *t-*test). Summary data are mean ± SEM. (C) Expressions of CXCR3 and CCR6 by the peripheral blood ILC subsets of normal donors and autoimmune liver disease patients with the condition autoimmune hepatitis (AIH). Summary data are median ± interquartile range. (* = p<0.05 by Mann-Whitney test).

We examined ILC2 for expression of the chemokine and integrin receptors CXCR3, CCR6, Very Late Antigen-5 (VLA-5) and Very Late Antigen-6 (VLA-6) that have been implicated in lymphocyte recruitment and positioning in inflamed diseased livers. Intrahepatic ILC2 had low-moderate expression of CXCR3 whose ligand, IP-10, was secreted by stimulated primary human biliary epithelial cells (BEC), stellate cells and fibroblasts (Fig 5B). The majority of liver-derived ILC2 expressed CCR6 (50–90%) (Fig 5A), which mediates homing of lymphocytes to inflamed bile ducts in response to CCL20 secreted by biliary epithelium [21]. We then explored the possibility of increased ILC2 infiltration in liver diseases by examining the expression of liver homing CXCR3 and biliary homing CCR6 chemokine receptors by peripheral blood ILC of normal and autoimmune liver disease patients. Disease state did not alter the expression of CXCR3 by any ILC subset in the peripheral blood but blood ILC2 had very low CXCR3 expression compared to ILC1 or ILC3 (Fig 5C). As in liver, we observed an increase in CCR6 expression by blood ILC2 in disease. This induction of CCR6 expression in the blood in disease was unique to the ILC2 subset (Fig 5C). In addition, intrahepatic ILC2 expressed high levels of the integrins Very Late Antigen-5 (VLA-5) and Very Late Antigen-6 (VLA-6) that bind fibronectin and laminin both of which are components of the fibrous stromal tissue that surrounds the bile ducts in portal tracts (Fig 5A).

### Intrahepatic ILC2 express IL-13 and amphiregulin

**Fig 6:**
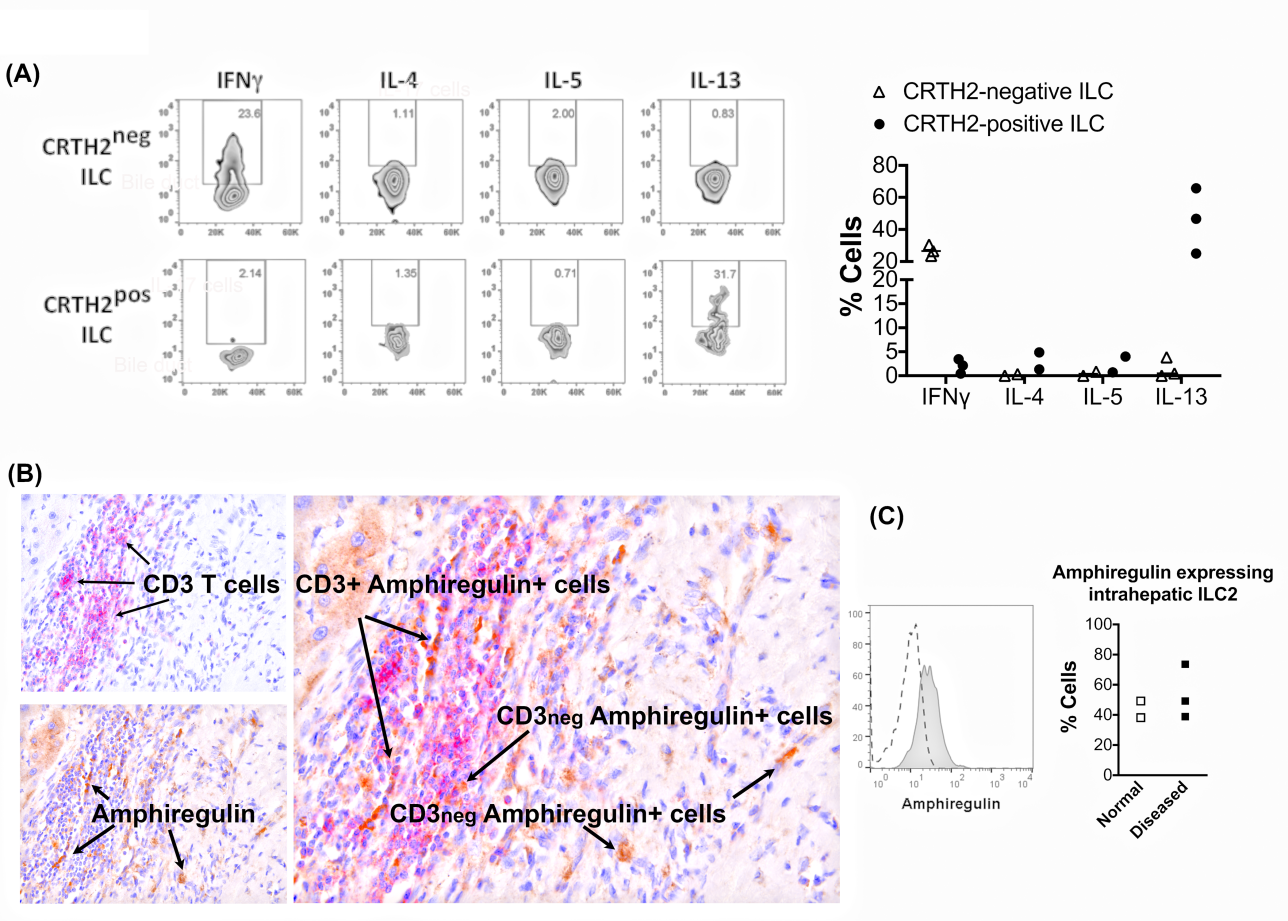
Biliary epithelial cells secrete IL-33 and intrahepatic ILC2 express IL-13 and amphiregulin. **(**A) Intrahepatic ILC were freshly isolated and stimulated with PMA and Ionomycin for 4 hours and both CD45^pos^ CD3^neg^ lineage^neg^ CD127^pos^ CRTH2^pos^ ILC2 and CD45^pos^ CD3^neg^ lineage^neg^ CD127^pos^ CRTH2^neg^ populations were analysed for expressions of IL-4, IL-5, IL-13 and IFN-γ. Representative dot plots and summary data are shown. (B) Immunohistochemistry for amphiregulin in CD3 positive and negative intrahepatic immune cells in human liver. CD3 (Vector Red, red color); Amphiregulin (DAB, Brown color). (C) Amphiregulin expression by intrahepatic ILC2. Amphiregulin representative overlay and summary data in normal and diseased livers are shown. In the histogram overlay the dotted line represents isotype staining and the shaded histogram reports amphiregulin staining.

Intrahepatic ILC2 secreted IL-13 but not IL-4 or IL-5 (Fig 6A). In contrast to ILC2, CRTH2^negative^ ILC (ILC1 and ILC3) secreted IFN-γ but no Th2 cytokines (Fig 6A). Given their close association with biliary epithelium we investigated if ILC2 secrete the tissue regenerative growth factor amphiregulin. We demonstrated by flow cytometry that intrahepatic ILC2 have constitutive expression of amphiregulin in the normal and diseased setting (Fig 6C). ILC1 and ILC3 subsets also expressed amphiregulin at variable levels (ILC1: 25–85% and ILC3: 40–60%). Amphiregulin was also detected on both CD3^pos^ and CD3^neg^ cells in tissue by immunohistochemistry (Fig 6B).

## Discussion

Innate lymphoid cells, which derive from common lymphoid progenitor (CLP) cells, are emerging as a family of effectors and regulators of innate immunity. They play beneficial roles in epithelial repair and in metabolic homeostasis. ILC1, ILC2 and ILC3 represent innate counterparts of Th1, Th2 and Th17 cells based on their cytokine secretion. Here, we report that all ILC subsets are present in the inflamed human liver including ILC1, prostaglandin D2 receptor CRTH2^pos^ ILC2 and both NCR^pos^ (NKp44^pos^) and NCR^neg^ (NKp44^neg^) ILC3 populations. In contrast to one recent report on ILC in the human liver[27], we identified that the ILC1 subset is the predominant ILC subset in both normal and diseased livers. Since a shift in the ILC balance toward reduced ILC1 predominance was seen in disease it seems likely that the maintenance of high ILC1 in the normal setting is a reflection of a role for ILC1 in immunosurveillance, maybe as a first line of defense against pathogens entering from the gut [28] [29]. Production of IFNγ by this subset agrees with such a role. There was a significant reduction in ILC1 in the autoimmune liver diseases and tendency for this was also seen in the non-autoimmune liver diseases. In contrast, the ILC2 and ILC3 frequencies showed evidence of increase with liver disease and ILC2 frequencies correlated with disease severity as measured by model of end stage liver disease (MELD) scoring leading us to conclude an ultimate pathogenic role for ILC2 and to focus our studies on the ILC2 subset [30]. The mechanism of increase in ILC2 is beyond the scope of the present study.

The expression of the liver-homing chemokine receptor, CXCR3, by intrahepatic and blood ILC2 supports the potential for infiltration by these cells to the liver. However since the expression of CXCR3 by blood ILC2 was low compared to by the ILC1 and ILC3 subsets it would be expected that ILC1 and ILC3 infiltration would be favoured over ILC2 if it were based only on CXCR3 expression. CCR6 mediates recruitment to CCL20 expressing cells of the liver, such as inflamed/infected biliary epithelium[21]; thus the significant upregulation of CCR6 on blood ILC2 in autoimmune liver disease, as an example of a liver disease suggested that additional chemokine receptors might provide a supportive signal for recruitment in disease.

ILC2 have been reported to have profibrotic and regenerative potential and have been identified at mucosal surfaces including the intestine, respiratory tract and skin where they orchestrate host interactions with the environment [31–33]. We found that intrahepatic ILC2 displayed a tissue resident phenotype evidenced by a high level of CD69 expression[34]. Consistent with previous reports from human gut and lung, they expressed the C-type lectin CD161 [7] which binds lectin like transcript-1 (LTT-1). Although its role is not known, CD161 has been associated with intrahepatic lymphocytes including MAIT cells and some effector subsets [35]. The CRTH2 receptor binds PGD_2_ [36–39], which is secreted by mast cells, recruits Th2 cells and eosinophils in allergic responses and is detected during hepatic fibrogenesis [40]. Activation of the PGD_2_-CRTH2 pathway has been reported in fibrotic diseases including interstitial fibrosis in the kidney [41]. We detected PGD_2_ in liver tissue which might influence ILC2 migration and cytokine secretion through CRTH2, since blockade of CRTH2 with Ramatroban inhibited the migration of naive Th2 lymphocytes [42] and the accumulation of ILC2 in the inflamed lung[32] and skin[8].

Intrahepatic ILC2 expressed the high affinity IL-2 cytokine receptor, CD25 and the IL-7 cytokine receptor, CD127. Both IL-2 and IL-7 are known survival cytokines for ILC2 [1]. We recently reported very low levels of IL-2 in human liver tissue and identified that the source of intrahepatic IL-2 was from activated intrahepatic CD4 T cells [43]. Thus it is possible that ILC2 respond to IL-2 secreted by co-located T cells at sites of inflammation with which they interact through MHCII [44]. Low concentrations of IL-7 enhance the survival of T cells [45] but it has been reported that ILC require a higher concentration of IL-7 along with Notch signaling for their survival (Shigeo Koyasu, personal communication). IL-7, however, was present only at low levels in liver tissue. On the other hand we were able to detect another ILC2 survival cytokine, IL-9, in inflamed liver tissue [46]. In contrast IFN-γ, which is known to inhibit the proliferation and function of ILC2 [26] is present at high levels in inflamed liver tissues [46] suggesting that on balance the inflamed intrahepatic microenvironment is likely to be hostile for ILC2.

ILC2 are crucial in epithelial barrier protection [47]. Intrahepatic ILC2 expressed the chemokine receptor CCR6, which we have shown to attract effector cells in response to the CCR6 ligand CCL20 secreted by biliary epithelium under proinflammatory conditions [19, 48]. Thus, it is likely that ILC2 may utilize the CCR6-CCL20 pathway to respond to alarmins produced by inflamed and injured BEC.

Emerging evidence suggests that ILC2 play a role in responses to bacteria through the production of type-2 cytokines such as IL-5 and IL-13 [49]. ILC2 and Th2 are responsive to epithelial cytokines [50]. Unlike their Th2 counterparts, ILC2 lack rearranged antigen specific receptors and cannot recognize antigen selectively but instead respond rapidly to bacterial cell wall products or alarmin cytokines released by damaged epithelium. One such cytokine is IL-33, which drives bile duct proliferation in children with biliary atresia [17] and is required for the development of hepatic fibrosis *in vivo* [15]. ILC2 also respond to IL-33 in immune-mediated hepatitis [30]. We detected low levels of IL-33 secretion by BEC and a very low frequency of intrahepatic ILC2 expressing the IL-33 receptor ST2 suggesting that IL-33 may not be a dominant survival signal for ILC2 in the inflamed human liver.

ILC subsets are one of the important sources of effector cytokines during tissue homeostasis and inflammatory conditions. ILC2 secrete IL-13 in blood [51] and IL-13 and IL-5 in respiratory mucosal tissue [11]. MHCII expressing ILC2 can interact with CD4 T cells leading to bidirectional signaling and production of IL-2 from T cells, which promotes ILC2 proliferation and IL-13 production [44]. We found that human intrahepatic ILC2 produce interleukin-13, but we could not detect IL-4, IL-5 or IL-9 secretion by these cells. The secretion of IL-13 is consistent with a profibrotic role [5, 15, 52].

ILC2 cells have been implicated in tissue remodeling [53] through an IL-33-amphiregulin EGFR dependent pathway [54]. Amphiregulin, a member of the epidermal growth factor family, leads to tissue regeneration via activation of the epidermal growth factor receptor (EGFR) [55] which is present on biliary epithelium [54, 56, 57] and acts via the Akt and STAT3 survival pathway. Pulmonary ILC2 secretion of amphiregulin [14, 54] restores the integrity of the airway epithelium following influenza infection [58]. Amphiregulin is detected in chronically injured human liver and can be rapidly induced following partial hepatectomy in rodents [59]. We detected amphiregulin by immunohistochemistry in human liver tissue. Staining co-localised with non-CD3 cells and we verified by flow cytometry the presence of amphiregulin expressing ILC cells including ILC2 in the human liver. Thus ILC2-derived amphiregulin could potentially drive regeneration and proliferation of bile ducts through EGFR activation and it is possible that local IL-33 signaling via induced ST2 expression could enhance ILC2-mediated fibrosis. Unfortunately, due to the small numbers of ILC2 that could be recovered from end stage liver tissue and the plasticity they demonstrate on *in vitro* expansion we were unable to evaluate the functional effects of ILC2 in co-culture with BEC [60].

**Fig 7:**
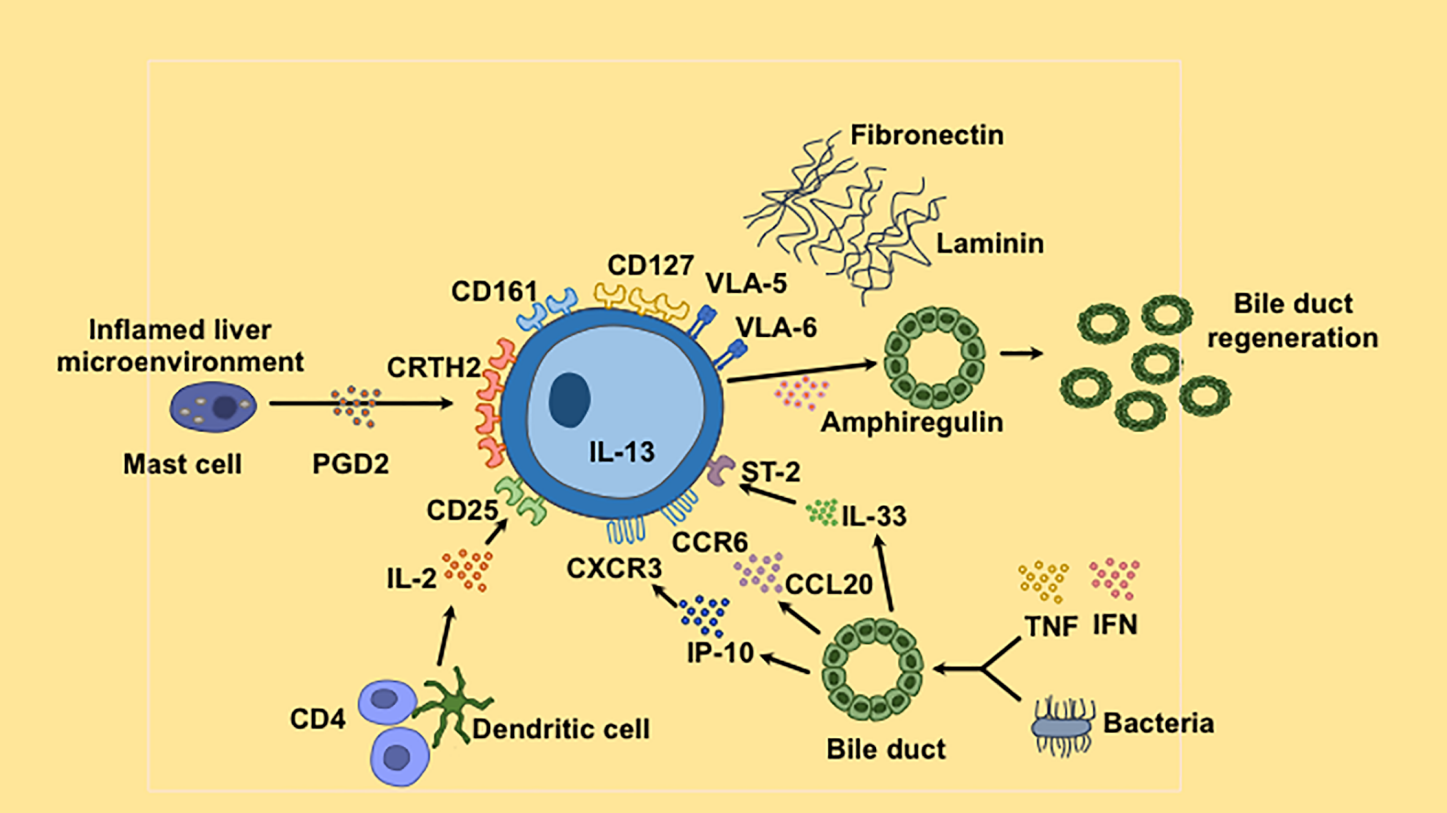
Summary graphical diagram. Intrahepatic ILC2 are characterized by expression of CD127 and CRTH2, which binds PDG2. They express tissue residence maker CD69; CD161; the IL-2 cytokine receptor CD25; liver and tissue homing chemokine receptors CXCR3 and CCR6 and cytokine receptor ST2, which bind respectively to IP-10, CCL20 and IL-33 produced by biliary epithelial cells upon stimulation with inflammatory cytokines or bacteria. ILC2 secrete IL-13 and amphiregulin, which may contribute to bile duct regeneration. In addition, they express the integrins VLA-5 and VLA-6, which attach to the extracellular matrix proteins fibronectin and laminin found in the fibrous stroma.

In conclusion, we report for the first time that relative intrahepatic ILC2 frequency increases with worsening liver function as assessed by the model of end stage liver disease (MELD) score in human liver cirrhosis. ILC2 may increase as an attempted protective response to repair tissue damage through IL-13 and amphiregulin, which might result in both fibrogenesis and biliary proliferation (Fig 7). The high expression of CRTH2 on ILC2 suggests that these cells could potentially be targeted with Cyclooxygenase-2 inhibitors to reduce their fibrogenic potential during the evolution of liver disease.

## Abbreviations

NL: normal liver
AILD: autoimmune liver disease
NASH: non-alcoholic steatohepatitis
ALD: alcoholic liver disease
ILC: innate lymphoid cells
CRTH2: chemoattractant receptor-homologous molecule expressed on Th2 cells
PGD2: Prostaglandin D2
